# Finding shared solutions in landscape or natural resource management through social learning: A quasi-experimental evaluation in an Alpine region

**DOI:** 10.1007/s10980-021-01274-y

**Published:** 2021-06-05

**Authors:** Matthias Buchecker, Marius Fankhauser, Raphael Gaus

**Affiliations:** Research Unit Economics and Social Sciences, Swiss Federal Research Institute WSL, Zürcherstrasse 111, CH-8903 Birmensdorf, Switzerland

**Keywords:** Social learning, Actors’ problem perspectives, Public participation, Integrated resource management, Repeated measurement, Strategic planning

## Abstract

**Context:**

The implementation of landscape-management decisions is often blocked because actors disagree in their perception of the problem at hand. These conflicts can be explained with the concept of problem framing, which argues that actors’ problem perspectives are shaped by their interests. Recent literature suggests that social learning through deliberative processes among actors enables shared solutions to complex landscape-management conflicts.

**Methods:**

To examine these assumptions, a participatory process on integrated water-resource-management in a Swiss Alpine region was systematically evaluated using a quasi-experimental intervention-research design. The involved actors’ problem perspectives were elicited before and after the participatory processes using qualitative interviews and standardized questionnaires. Furthermore, a standardized survey was sent to a sample of regional residents (N = 2000) after the participatory process to measure the diffusion of actors’ social learning to the wider public.

**Results:**

The data analysis provided systematic evidence that a convergence of involved actors’ problem perspectives, which were found to differ considerably before the intervention, had taken place during the participatory process. Furthermore, it determined diffusion effects of actors’ social learning to the wider public in terms of its attitude towards participatory regional planning.

**Conclusions:**

The findings confirm the expected mechanism of social learning through deliberative processes and demonstrate it as a promising approach to implementing landscape-management decisions successfully. The catalyzing role of shared interests among actors suggests that landscape-management decisions should be implemented by participatory integrated planning on the regional level, which would require a new, strategic role of regional institutions.

## Introduction

### Implementation gap in environmental planning

Against high expectations, the implementation of environmental policies in Europe such as the Water Framework Directive or the Swiss Water Protection Act have shown limited progress (Voulvoulis et al. 2017). River revitalizations are a distinctive example for the gap between societal decisions and implementation. The Swiss Water Protection Act, which was enacted in 2011, stipulates that structural flood protection measures should always involve ecological enhancement of the river environment. In many cases, however, opposition by local farmers, property owners, or authorities of affected municipalities result in a reduced implementation of the planned ecological enhancements (Menzel and Buchecker 2013; Verbrugge et al. 2019). Planners and members of state agencies tend to explain opposition to environmental measures with opponents’ lack of knowledge of the problem they consider to have been objectively given (Demeritt and Nobert 2014). Accordingly, they call for information campaigns or instrumental participation to raise awareness and build acceptance among opponents (Buchecker et al. 2013).

### The challenge of problem frames

**Fig. 1:**
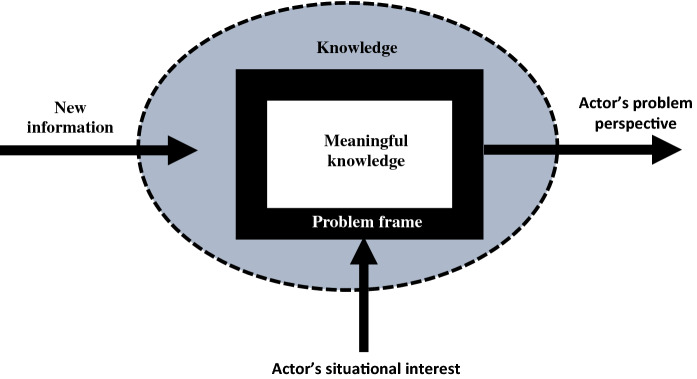
Conceptual model of actors’ problem perspective in a conflict or decision situation (according to Kolkman et al 2007 and Gaus et al 2020)

However, recent studies highlight that most environmental problems in modern societies are characterized by a high ambiguity, which means that the affected actors have their specific problem perspectives (Kolkman et al. 2007). This can be explained by the concept of problem framing according to which an actor’s problem perspective is not only informed by the information on the problem aspects they have received and adopted, but is also shaped or framed by their situational interest (Fig. 1) (Kolkman et al. 2007; Asah et al. 2012; Gaus et al. 2020). This concept suggests that the more conflicting the situation, the more the actors tend to protect their interests and thus narrow down their problem perspective. Problems that are characterized by high ambiguity can therefore not be solved by forms of participation that focus only on acceptance building, and they can even less be enforced without substantial social damage (Menzel and Buchecker 2013). More deliberative forms of participation are required (Renn et al. 2011). Moreover, theoretical literature suggests that shared solutions to complex environmental problems or conflicts can only be found through social learning processes that allow actors to reframe their problem perspectives and thereby increase the space for shared solutions (Biggs et al. 2011). According to this understanding, solutions are integrative parts of actors’ problem perspectives rather than contingent options, such as in the approach by Gerrits and Marks (2017).

### Social learning as a promising loophole

**Fig. 2:**
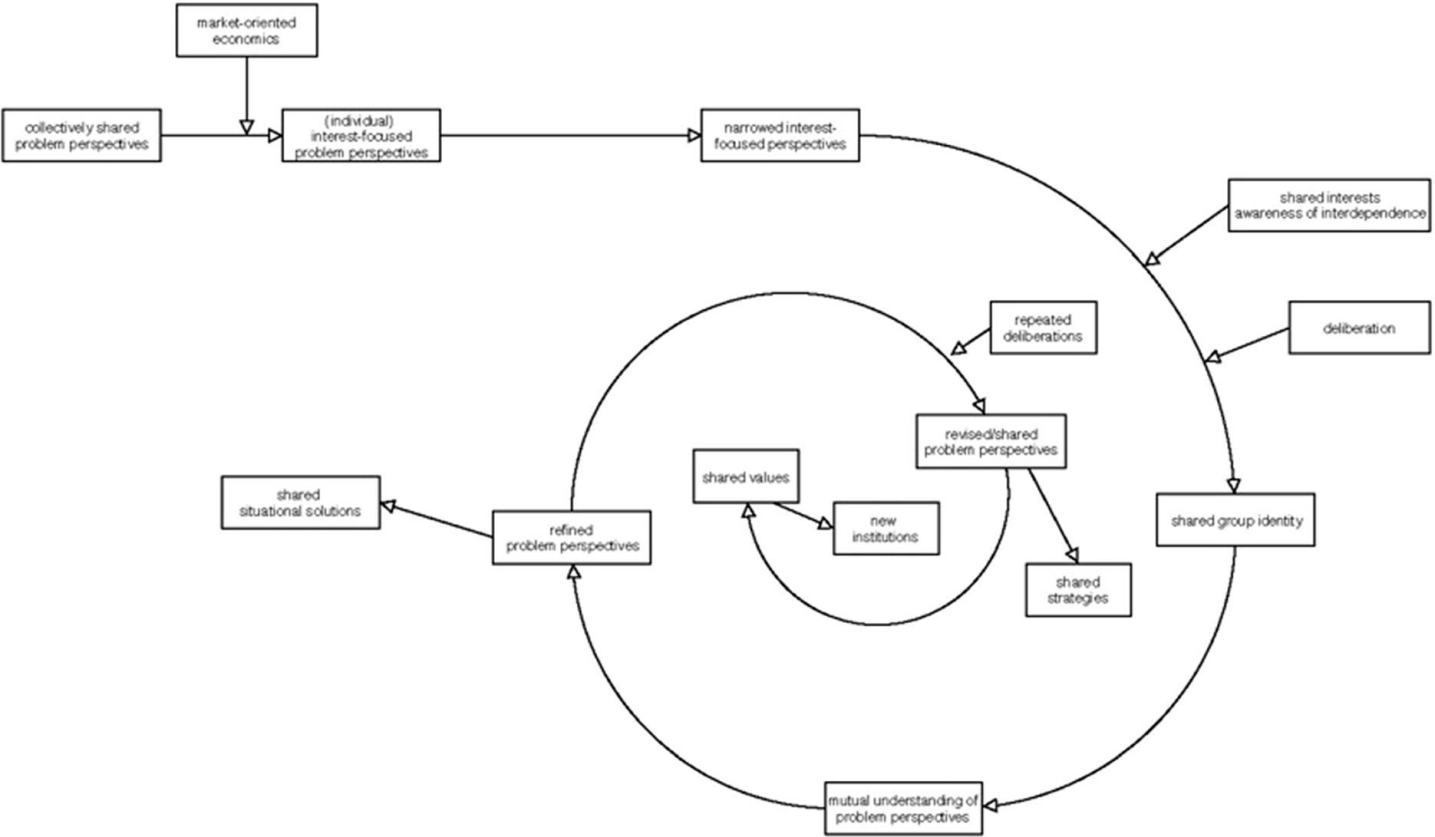
Framing and reframing of actors’ problem perspectives in a social learning process. The boxes integrated in the loop indicate expected stages of the learning process, the inbound arrows stand for conditions expectedly needed for the promotion of the learning process, and the outbound arrows denote expected outcomes of the learning process

Early definitions of social learning go back to Bandura (1977) who emphasized that individuals learn by observing the behaviors of others. A more recent approach that has emerged in the context of research on natural resource management suggests that social learning implies group and deliberative learning (Finger and Verlaan 1995; Pahl-Wostl et al. 2007; Armitage et al. 2008). There is, however, no general agreement on the key aspects of this concept so far and a wide range of contrasting assertions about the process and the outcomes of social learning exists (Reed 2008; Garmendia and Stagl 2010; Cundill and Rodela 2012). According to Muro and Jeffrey (2008), who elaborated a coherent framework based on extensive literature review, social learning: (i) is enabled by communication and interaction in participatory processes; (ii) includes recognizing each other’s goals, perspectives, and underlying values; (iii) leads to acquisition of factual knowledge and skills, change of cognitions and attitudes and improvement of trust and relationships; and (iv) contributes to common understanding, mutual agreement, and collective action. Recent studies furthermore pinpointed that social learning leads to a convergence of actors’ perspectives on discussed issues, to improvement of relationships, and greater group identity (Biedenweg and Monroe 2012). This creation of group identity plays a crucial catalytic role in this learning process: in particular, the opening of actors’ problem frames and finding shared solutions (Cundill 2012; Schusler 2003). Literature on common pool resources (Ostrom et al. 1999) additionally highlight that building a strong enough group identity among actors to overcome problem frames based on specific interests requires that actors are aware of their interdependence and the existence of shared interests. Therefore, Ostrom et al. (1999) sees the best potential to find shared solutions in natural resource management on a regional level, where actors can experience their interdependence in their daily practice. Finally, literature on organizational learning suggests that, depending on the intensity and quality of interaction among actors, different degrees or depth of social learning takes place. Single loop learning refines and opens actors problem frames, and allows for shared situational solutions. Double loop learning questions actors’ problem understanding in a more generalized sense, thereby facilitating collaborations among actors. Triple loop learning questions the actors’ beliefs and values, and provides the basis for new forms and institutions of collaboration (Argyris 1999, 2005; Peschl 2007; Biggs et al. 2011; Eriksson et al. 2019). In particular, repeated and more dialogic participatory processes seem to promote social learning to a greater extent (Muro and Jeffrey 2008; Leach et al. 2013; Ernst 2019). Figure 2 depicts this complex process in the form of a helix that expresses the looped character of the learning process (Biggs et al. 2011). Although the conceptual basis of social learning has been established for nearly a decade, there is so far only little, and in particular little systematic, evidence for the effectiveness of social learning in real decision making. With the study presented in this paper, we evaluated a real participatory decision process in order to evaluate the extent to which social learning through deliberative processes among actors really takes place and enables actors problem frames to be overcome. In particular, we examine the following research questions:

- How much do actors’ problem perspectives differ?
- Do regional actors have shared interests?
- How much do actors’ problem perspectives open or converge during a participatory process, and on which level?
- Which other outcomes of the learning process could be determined?
- How much do actors’ problem perspectives converge through information about the participatory process alone?
- To which extent does the evaluated process correspond with the expected social learning mechanism?

## Effectiveness of social learning

The diversity of definitions and conceptual assumptions in the rapidly growing literature on social learning has hindered systematic evaluation (Cundill and Rodela 2012; Johannessen and Hahn, 2013). A number of explorative studies that were based on diverse theoretical frameworks have confirmed that social learning is facilitated by regular social interactions and, in particular, by deliberative processes among actors (Garmendia and Stagl 2010; Leys and Vanclay 2011). A large European study based on descriptive case study comparisons found that participatory river projects could contribute to actors’ social learning in terms of conflict reduction, trust building, implementation of projects, or institutional changes, if the exchange of actors’ problem understandings was accommodated in the process (Mostert et al. 2008). A qualitative accompanying evaluation of a research conference on local resource management revealed that most participants agreed on a common purpose by achieving a greater understanding of the issue and recognizing the interests of other participants as legitimate (Schusler et al. 2003). Furthermore, a qualitative analysis of three extensive workshops, in India, Brazil, and Mali, on natural resource management involving local and external actors, found consistent evidence that deliberative knowledge exchange substantially increased the learning process and enabled the transfer from a sustainable management to a sustainable governance of natural resources (Rist et al. 2007). After a first phase of trust building and establishing informal communication, the participants started to change the mutual perceptions of local and external knowledge, which led them eventually to reflect and question the norms and rules of the use of natural resources. Furthermore, a qualitative ex-post evaluation of five completed participatory river revitalization projects found that actors perceived social learning in terms of attitude changes towards the integrative river management as well as improved competences in how to find a consensus were the most relevant benefits of the participatory processes: even more relevant than trust or acceptance building (Menzel and Buchecker 2013).

Early empirical studies using more systematic (repeated) measurements provided some evidence that actors enhanced their understanding of other groups’ positions during small sized participatory processes and achieved a better consensus on future developments and measures (Borowski et al. 2008; Buchecker et al. 2010; Garmendia and Stagl 2010; Albert et al. 2012). There is, however, a lack of empirical evidence as to whether discursive processes in risk or resource management really lead to a shared understanding of the problem or issue rather than a superficial ad-hoc consensus (Muro and Jeffrey 2008). In accordance with, Fischer (2003), Reed (2008), Pahl-Wostl et al. (2008), van Bommel et al. (2009) and Ernst (2018), we consider this convergence of actors’ problem understanding to be the most critical aspect of social learning. Recently, cognitive methods for eliciting problem perspectives have been recognized as a systematic technique for identifying subjective perspectives and measuring their convergence (Biedenweg and Muroe 2013).

## Effects of deliberative processes on actors’ problem understanding

Recent theoretical literature highlights the effectiveness of deliberative processes in not just facilitating a change of actors’ understanding of risk and resource management problems, but also in achieving a shared problem understanding, (Pahl-Wostl et al. 2008; Mostert et al. 2008; Gamendia and Stagl 2010; Biggs et al. 2011). There is, however, little and inconsistent empirical evidence whether, under which conditions, to what extent, and through which mechanisms, deliberative processes can enable a substantial convergence of actors’ problem understandings (Muro and Jeffrey 2008; Leys and Vanclay 2011; Cundill and Rodela 2012). Until recently, empirical evidence that social learning leads to shared perspectives has been limited to qualitative methods (Biedenweg and Monroe 2013). Consequently, the potential of communication to change problem understandings, and deeply entrenched beliefs in particular, is doubted by a number of authors (Irwin 2006; Terpstra et al. 2009; Kahan et al. 2011). Kolkman et al. (2007) observed, in a qualitative ex-post study in which they reconstructed the mental models of actors involved in a decision process on integrated water management based on interviews and document analysis, that open dialogue among all main actors did not lead to a convergence of the actors’ mental models on the management problem. Similarly, an evaluation of a multi-actor negotiation process based on media analysis, participatory observation, and qualitative interviews resulted in the conclusion that no convergence of actors’ ideas, in terms of goals and means to deal with the problem, had taken place (van Bommel et al. 2009). The lack of perceived interdependence and unequal power relationships were identified as the main reasons for the failure of social learning. Gray (2004), in her analysis of a participatory process, found that differences in actors’ problem frames that were based on strong group identities could not be overcome or reframed. Heeb et al. (2008), however, could demonstrate in their descriptive study that a long-standing conflict between hunters and foresters, in the context of the management of an avalanche protection forest, could be solved through a process of dialog in which the stakeholders mutually exchanged their problem understandings based on concept mapping (a technique to visualize mental models). Similarly, Lane et al. (2011) found, in an action research study, that involving lay expertise in participatory modelling of a flooding problem enabled the group to reframe the problem and to devise new solutions. Some (small-scaled) quantitative evaluations of deliberative processes, based on the mental model and concept frame approaches, have provided more robust evidence that deliberative processes can contribute to single, or even double,-loop learning. Quantitative ex-post evaluations of participatory concept mapping workshops on environmental problems, which were conducted in game settings, have confirmed that collectively reflecting on problem understandings has an enhancing and clarifying effect on shared problem definition (van Kouwen et al. 2009). A more systematic approach to identifying whether deliberative processes can promote a shared problem understanding was developed and tested by Mathevet et al. (2011). Based on a comparative mental model analysis, they could provide evidence that the more frequently stakeholders had attended meetings of a non-statute Water Board, the more their mental models of the management problem overlapped. Moreover, Biedenweg and Monroe (2013) could provide some quantitative evidence that members of social learning groups had more similar mental models of ideal community forest management than those who had not participated in an interactive learning process. In their study, they explored and compared the content and structure of local inhabitants’ knowledge using the method of conceptual content cognitive mapping and consensus analysis. They, however, admitted that their findings were not conclusive in determining whether the convergence of perspectives was a result of a knowledge transfer imposed by powerful participants or a genuine knowledge exchange. Finally, Ernst (2018) conducted a comprehensive retrospective self-reporting survey, which was sent to a representative online panel of German people who had participated in a planning or decision-making process related to energy transition. The results showed that more than 40% of participants reported to have changed their attitudes about problems related to energy transition and 23% reported that their attitudes had been disproved (value change) by the participation process Ernst (2018).

The literature review shows that there is a lack of robust evidence, based on systematic research, as to whether and to which extent (single, double or triple-loop learning) deliberative planning in risk and natural resource management can contribute to a convergence of the actors’ problem understanding (Khadka et al. 2013; Biedenweg and Monroe 2013; Medema et al. 2015; Ernst 2018). It is furthermore unclear how the social learning effects diffuse to the wider community (Schusler et al. 2003; Cundill and Rodela 2012). Finally, the role of the relationship between the actors, and in particular the development of a shared identity among actors, for social learning has not yet been systematically considered.

## Case study

To answer our research questions, we systematically evaluated the processes in a participatory integrated water resource management project: Gewässerentwicklungskonzept (GEK) Hasli, in the Hasli valley in the central Swiss Alps. The goal of this participatory process was to elaborate a vision for the future of the regional water bodies, to define technical and institutional measures to manage these water resources, and to launch integrated river management projects. The format of the GEK Hasli project is novel in Swiss water resource management but had been tested in a less comprehensive form in two other river basins in the Canton of Berne. This format combines a legal procedure: the strategic regional river management planning as defined in the Cantonal Act on Water Management (WBG 1989), with a more informal procedure of participatory visioning (Hatzilacou et al. 2007) focusing on regional water resources.

The participatory process was commissioned and funded by the water management agency and fishery agency of the Canton of Berne. It included 10 deliberative workshops that were conducted between spring 2015 and autumn 2017 with overall 62 participants. The project team was managed by a professional planner and moderator and led by a member of the fishery agency who purposefully selected the participants based on systematic stakeholder identification. The sample of invited participants included members of all relevant regional actor groups including local administration, local water management, agriculture, energy production, regional transport, local nature protection, fishery and regional tourism along with members of relevant cantonal and federal agencies (water management, agriculture, energy production, nature, and landscape protection).

**Fig. 3:**
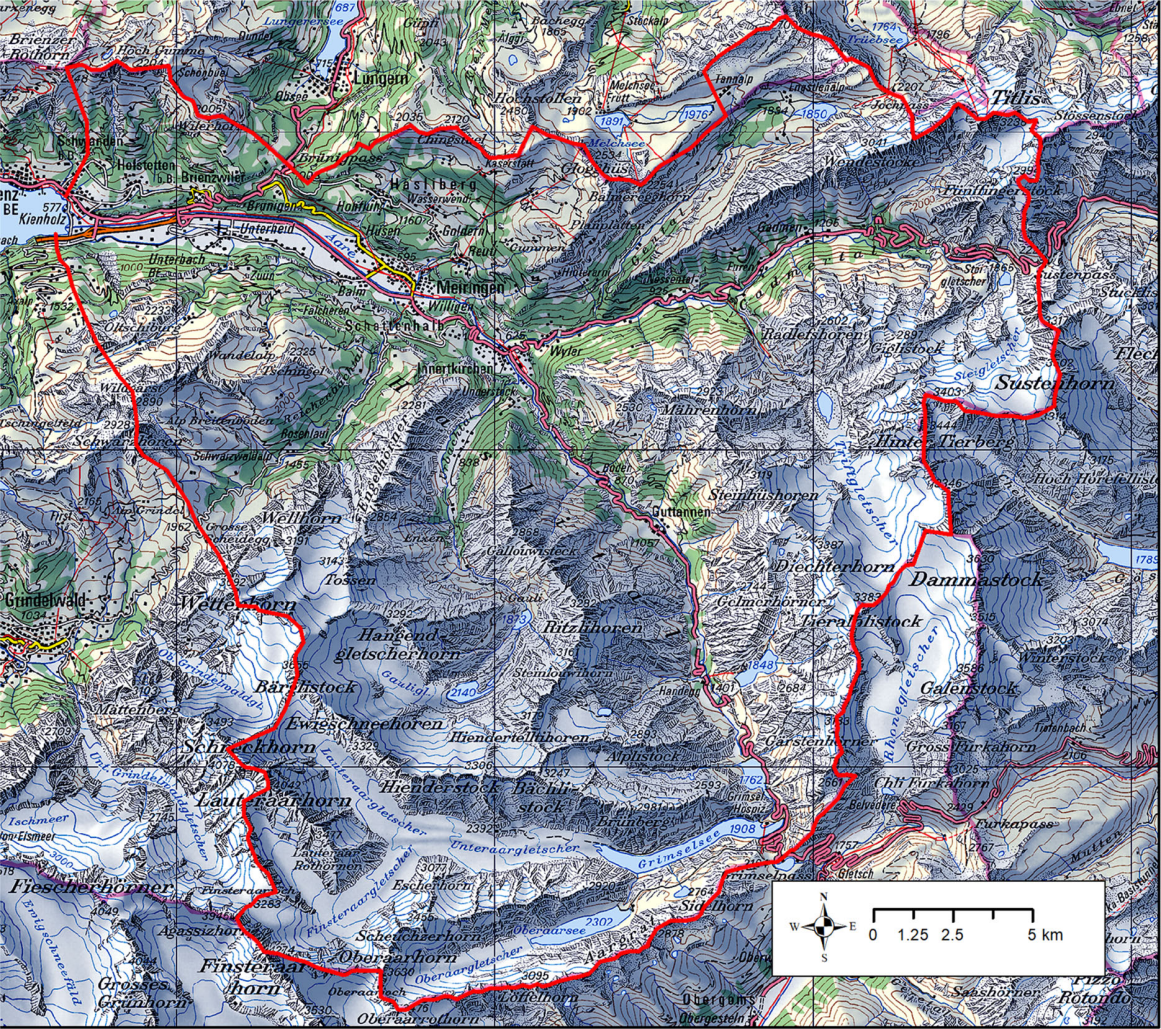
The perimeter of the GEK Hasli (red line). Area colors: green = forests; white = glaciers; blue = (barrier) lakes

The project perimeter of the Hasli valley covers an area of about 600 km^2^ with some 12 500 inhabitants domiciled in nine municipalities (Fig. 3). Hasli valley is the name of the uppermost catchment area of the Aare rivers including glaciated high mountain areas; wild, deeply incised upper mountain valleys; and a broad, originally waterlogged trough valley that ends in the Brienzer lake. This Alpine region had traditionally been important because of its transit routes to the south and east before becoming a tourism center in the belle epoch, and receiving a key role in hydro energy production in the early twentieth century. The region has been considered an icon of natural hazards since a series of extreme events in the late twentieth century. This multitude of functions concentrated in a narrow valley has brought about a number of serious conflicts in the past and provides maximally challenging conditions for an integrated resource management.

## Methods

### Research strategy

**Fig. 4:**
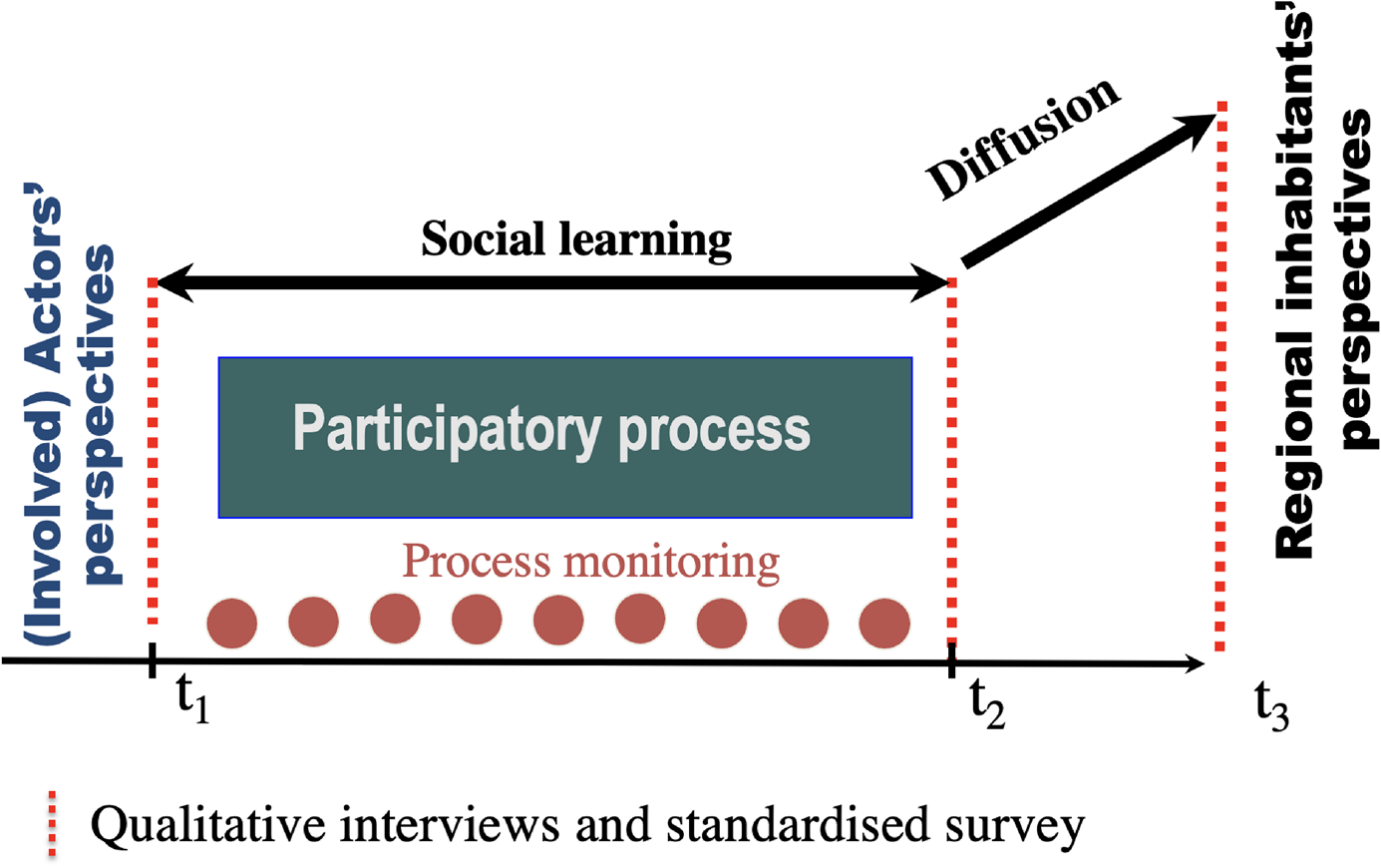
Research design of the study

In this quasi-experimental intervention research (Buchecker et al. 2010), the effects of the deliberative planning process on involved actors’ social learning were elicited by repeated measurement. Furthermore, the diffusion of the social learning effect to the regional population was quantified by an additional standardized cross sectional survey (Fig. 4).

This design of quasi-experimental intervention research allows the researchers to go beyond simply confirming correlations between actors’ involvement in social interactions and the similarities in their understanding of complex management problems (such as in Mathevet et al. 2011) by also reliably revealing the direction of effects. In environmental psychology, field-experimental intervention research has been developed and applied using various forms of interventions aimed at changing attitudes to environmental issues and related behaviour (e.g.Dwyer et al. 1993; Mosler and Tobias 2000).

As a deliberative process can be considered to be a type of intervention, it can also be evaluated with a similar kind of experimental design, but with two major differences concerning the design of the experiment. One difference is that this kind of intervention primarily has an effect on the social, and only indirectly on the physical, environment, which means that the effect cannot be determined, at least in the short term, with objectively measurable environmental data but rather with quasi-objectively measurable changes in people’s attitudes. The second difference is that, in a more principal sense, a “clean” comparative measurement of a control group within the same context is logically not possible for such interventions. This is, however, not problematic as the measurement of the effect is unlikely to be influenced by exterior disturbances given the relatively short time of the process and the focus on an issue of mainly local meaning.

### Data collection

A mixed method approach was used to measure social learning in actors and regional population, with the measurement focused on the change of respondents’ problem perspectives (Kolkman et al. 2007).

#### Inductive phase:

In a first step, qualitative interviews were conducted with 22 members of actor groups that were expected to be involved in the forthcoming participatory process. The interviews followed a semi-structured guideline and focused on interviewee’s perspective of the water management-related problem and their attitudes towards the participatory process for the integrated regional water management. The findings of this inductive phase have been described elsewhere by Gaus et al. (2020) and are treated here as a methodological basis for the standardized measurement of actors’ social learning. This qualitative pre-study revealed the three key elements that defined actors problem perspectives: the meanings (in the sense of perceived functions) the actors associated with water environments, the objectives the actors considered relevant for future regional water resource management, and the beliefs actors mentioned in the context of this issue.

#### Pre-measurement survey:

In a second step, we developed a standardized questionnaire to elicit actors’ perspectives on the issues related to the regional water resource management: drawing on the findings of the qualitative interviews. In particular, we asked the respondents to rate the relevance of meanings of the regional water bodies, the importance of objectives for their management, and their agreement with statements embodying beliefs and values related to regional water management based on a 7 point Likert scale (see detailed items in Table 2). In the following questions, the respondents were encouraged to assess their level of knowledge about diverse themes related to integrated water management; mark areas within the project perimeter with specific need for action on a topographical map; rate statements expressing attitudes towards the forthcoming participatory process; and indicate the degree to which they agreed with the attitudes and interests of the involved actor groups. In a final section, the respondents declared their foreseen role in the upcoming process and indicated their socio-demographic details including profession, age, length of residence, and place of residence.

The questionnaire was mailed to the actors immediately after the kick-off workshop (summer 2015), in which the actors were informed about the regional integrated water resource management and the participatory process. Due to the restrictions of the project team, the standardized questionnaires could only be sent to the 44 participants who had attended the kick-off workshop of the participatory process. To include members of the actor groups who were indirectly involved, we enclosed additional questionnaires with the letter and asked the addressees to distribute them to further members of their groups. In total, we received 50 completed questionnaires: 22 from directly involved actors and 28 from indirectly involved members of the actor groups.

#### Post-measurement survey:

In a third step, the questionnaire for the pre-measurement was adapted and complemented so that it could be used for the post-measurement of actors’ problem perspectives and to reveal actors’ assessment of the participatory process. The actors showed a certain reluctance to complete paper work so the number of items measuring the three elements of actors’ problem perspectives was reduced. The selection of items to be removed was based on a factor analysis of the variables measuring the single elements. In a similar manner, the items measuring actors’ knowledge about diverse themes related to integrated water management were reduced. Questions about actors’ roles and attendance during the participatory process were added to the questionnaire, as well as questions about the assessed quality of the participatory planning including the significance of quality criteria.

The standardized questionnaire was mailed to the involved actors immediately after the final workshop (summer 2017). Only few participants were replaced by other members of their actor group so the sample remained virtually the same. Again, additional questionnaires were enclosed with the letters to be distributed to other member of the actor group. Forty six respondents completed the questionnaire: 32 directly involved actors and 14 indirectly involved members of the actor groups.

Most of the respondents indicated their names on both questionnaires so a comparison on an individual level was possible. However, only 35 respondents filled in the questionnaire twice because 11 respondents who completed the second questionnaire did not belong to the sample of the first questionnaire.

#### Cross-sectional population post-survey:

To measure the diffusion of the actors’ social learning through the participatory process on the regional population, we elaborated a cross-sectional standardized questionnaire. As the project team did not agree to conduct a population survey related to the regional integrated water resource management, this questionnaire aimed to measure the diffusion of actors’ social learning by revealing the relationship between respondents’ problem perspectives and their information-level related to the process and outcome of the participatory process, while controlling for potential intervening variables. The standardized questionnaire was developed based on a number of qualitative interviews with regional residents and the pre- and post- surveys for measuring actors’ problem perspectives. in addition to a reduced set of items used for measuring the elements of actors’ problem perspectives in those surveys, the questionnaire included items on residents’ social integration in the region; their perception of, and attitudes towards, public involvement in regional decision making; their use of information channels to become informed about regional decisions; their information-level on the regional integrated water resource management process; their level of knowledge about aspects of regional resource management; their perceived agreement with interests and attitudes of involved actor groups; the assessed quality and added value of the integrated regional water resource management process; and their socio-demographic characteristics. Except for the latter and the variable on respondents’ information-level (five-point scale), all items were assessed on a 7-point Likert scale.

The standardized questionnaire was mailed to the households of the five municipalities of the upper Hasli valley nearly a year after the final workshop: a period that was long enough for communicating the process and short enough to maintain its local relevance. Questionnaires were sent to all of the households in the municipalities: Guttannen, Innertkirchen, Schattenhalb, and Hasliberg and a random sample of 550 households were selected in the municipality of Meiringen due to its large number of inhabitants (Table 1). In spite of the rather demanding character of the questionnaire, some 17% of the contacted households (342 respondents) sent back a fully completed questionnaire. The return rate was considerably higher in Meiringen where the respondents had been addressed personally with their name rather than collectively (as residents of the municipality) as was the case in the other municipalities.

**Table 1:** The sampling for the cross-sectoral standardized survey of the regional residents of the upper Hasli valley

Municipality	Inhabitants	Households	Sampling	Sample	Respondents	Response rate
Guttannen	267	120	All households	127	13	10%
Hasliberg	1193	486	All households	534	69	13%
Innertkirchen	1087	496	All households	532	73	14%
Meiringen	4692	2145	Random sample	550	120	22%
Schattenhalb	585	256	All households	250	31	12%
Not stated					36	
**Total**	**7824**	**3503**		**1993**	**342**	**17%**

Bold highlights the overall numbers of items, summing up the number of e.g. the inhabitants of all municipalities in the region

### Analysis of the data

The data of the standardized surveys were imported into an SPSS database, edited, and subject to descriptive statistical analysis. In a second step, a typology of actors with specific problem perspectives was conducted by principal component analysis using the 16 variables that measured respondents’ assessed degree of agreement with specific actor groups. This analysis was driven by the assumption that, in decision or conflict situations, actors tend to build few groups with similar conflict positions resulting in a limited number of actor types with conflicting problem perspectives (Asah et al. 2012). As the samples of the three surveys were only partly identical, aggregating respondents to actor types with very similar problem perspectives provided the opportunity to build and compare so called pseudo panels (Bernard et al., 2011). This procedure is particularly appropriate to illustrate and pinpoint shifts in conflicts.

The PCA revealed three components to which all the variables, with the exception of the variable related to the local water management corporations, clearly loaded (see Table 8 in the appendix). These components represent three actor types with problem perspectives focusing on the topics nature protection, agriculture, and hydropower. The thematic focus of the three actor types was substantiated by the analysis of the qualitative interviews from which the three themes of conflict emerged (Gaus et al. 2020). The unspecific loading of the variable related to water management corporations can be explained by the fact that these corporations include members from actor groups with contrasting interest positions: agriculture and the building industry. Accordingly, respondents belonging to these corporations were assigned to the types: agriculture or hydropower, depending on their profession, whereas the remaining respondents were assigned to the three types based on their primary actor group affiliation.

In a next step, actors’ social learning was quantified by comparing actor types’ changes in their problem perspectives using ANOVA.

In a final step, the diffusion of actors’ social learning was determined by a hierarchical regression analysis. In this analysis, the influence of respondents’ level of knowledge related to the regional integrated water management process on their attitudes towards ecological objectives and the success of this process were determined. As the respondents’ level of knowledge related to the regional integrated water management process was expected to depend on their social integration, which is likely to influence respondents attitudes towards public issues, all relevant variables measuring respondents social integration were included in the hierarchical regression to control their effects.

## Results

### Actors problem perspectives before the participatory process

The pre-measurement of actors’ problem perspectives show that the three actors types have a quite similar understanding about the meanings of regional waters, with four of 11 items differing significantly, but without any reversed value assignments (Table 2). A number of strong disagreements were found between the perceived assessment of objectives and beliefs related to the regional water management by the actor types. Regarding the actor types’ assessed relevance of objectives, only four of 12 items differed significantly, but with river restoration and expansion of hydropower production exhibiting reversed value assignments (< 4 vs. > 5). As for actor types agreement with beliefs. However, more than two thirds of the items to evaluate agreement with beliefs differed significantly between actor types, and most of these items included reversed value assignments. Conversely, all three elements assessing problem perspectives contained strong agreements or shared interests between actor types: in particular those related to flood and mud flow protection.

**Table 2:**
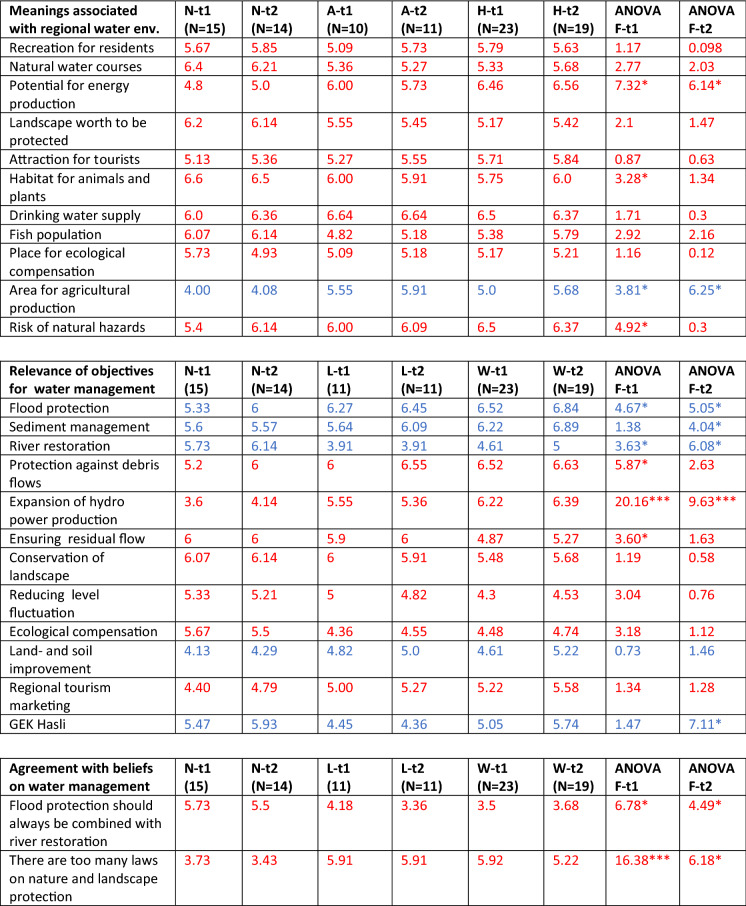

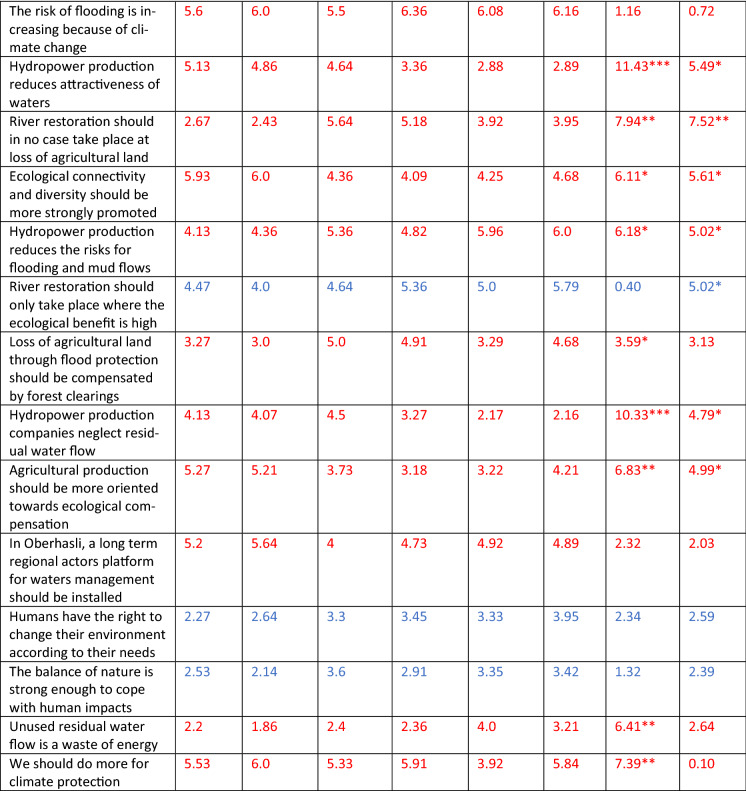
Actor types assessments of meanings related to regional water environments, objectives and beliefs related the regional water resource management before (t1) and after (t2) the participatory process

Scale: 1 = very irrelevant, 4 = neutral, 7 = very relevant

Colors: red indicating a decrease of group differences, blue an increase

*N* Nature protection actor type, *A* Agriculture actor type, *H* Hydropower actor type

*p < 0.05; **p < 0.005; ***p < 0.001

### Change of actors’ problem frames during the participatory process

The comparison of actor types’ problem perspectives before and after the participatory process highlights that considerable, and mainly positive, changes in the sense of ecological values have taken place (Table 2). Interestingly, the least changes took place regarding the element of meanings of waters. Most of the items showed only marginal changes in their mean values, with the most substantial change being the increased awareness of the natural hazard problem by the nature protection type respondents. Moreover, the differences between actor types’ ratings of meanings decreased for most items, and only two of the four differences remained significant.

More substantial changes were found in items indicating actor types’ assessment of objectives, with one item (Expansion of hydropower production) changing from a reversed to a neutral value assignment. Thereby, several changes towards more ecological values and higher risk awareness could be determined. At the same time, the differences between the assessments made by different actor types decreased for most of the items: indicating a convergence, in particular in terms of hydropower related aspects. The number of items with significant differences, however, remained the same.

Substantial changes of items indicating actor types’ agreement with beliefs also occurred, with one item: ‘we should do more for climate protection’, changing from a reversed to a positive value assignment () and one from a neutral to a negative value assignment. Thereby two tendencies could be observed: for a few items a polarization took place (in particular related to nature protection—agriculture conflicts), while for most items (in particular related to nature protection—hydropower production) a convergence of positions took place. Overall, the group differences decreased for most items during the participatory process, and also the items with significant differences decreased from 11 (before) to nine after the participatory process. Interestingly, the assessment of the two more generalized beliefs or values also changed substantively although not significantly.

### Actor types’ assessment of personal social learning

Against the results on actor types’ change of perspectives during the participatory process, their subjective ex-post assessments of their personal learning during the process was less positive (Table 3). All actor types indicated that they have moderately increased their knowledge about water resource management, and they were particularly positive about having got to know other actors’ attitudes and concerns. Most of the respondents of all actor types, however, quite clearly expressed that they have hardly changed their attitudes and concerns during the process, and in particular have not questioned their values related to water resource management. All actor types did, however, express a tentative change in their attitudes towards other actor groups.

**Table 3:** Actors ‘ ex-post assessments of their personal learning according to the three actor types

Which effects had the GEK on you personally	Actor Type Natur protection (N = 14)	Actor Type Agriculture (N = 11)	Actor Type Hydropower (N = 20)	ANOVA F-value
I have learnt new things about the water resources in Oberhasli	4.43	5.09	4.85	0.53
I have learnt new things about the procedure of water resource management	4.86	5.36	5.05	0.37
I have got to know new attitudes and concerns of the GEK participants	5.0	5.0	5.5	0.63
I have changed my attitudes and concerns related to the development of the regional water resources	2.79	3.73	3.5	1.15
I have changed my value orientation towards regional water resources	2.36	3.45	3.2	2.04
I have changed my attitude towards other actor groups	3.07	3.82	3.8	1.02
I have increase my confidence in the national and cantonal agencies	4.5	3.82	4.25	0.49
I have increased my confidence in local actor groups	3.86	4.73	4.8	2.06
I have established new contacts	4.57	4.82	5.1	0.64

Scale: 1 = does not apply at all, 4 = neutral, 7 = fully applies

### Actors types’ assessment of the success of the GEK Hasli

Actors’ ex-post assessment related to the success of the GEK Hasli process show that the three actor types consider all measured aspects of the process as moderately positive (Table 4). The most positive values were given to the items: ‘shared understanding of the problem” and “launched concrete measures’. Furthermore, the actors’ group identity was considered to be clearly positive. Interestingly, actors’ sense of belonging together appeared to be slightly higher than actors’ sense of ownership of the process: in particular in responses from the agriculture type. The only significant differences in actor types assessments were found regarding the item: ‘clear value added of the GEK’, which was rated more skeptically by the agriculture type.

**Table 4:** Actors’ ex-post assessment of the success of the GEK Hasli according to the three actor types

How do you assess the success of the GEK Hasli?	Actor type Nature protection (N = 15)	Actor type Agriculture (N = 11)	Actor type Hydropower (N = 20)	ANOVA F-value
*Content criteria*				
The GEK has promoted constructive solutions	5.77	5.09	5.35	0.87
The GEK has launched concrete measures	5.62	5.09	5.65	1.13
The GEK has promoted unwishful results	3.23	3.55	3.20	0.21
The value added of the GEK is unclear	2.08	3.73	2.70	5.49*
The GEK has improved a convergence of actors attitudes	5.38	5.09	5.30	0.21
The GEK has increased a shared understanding of the solutions to the problem at issue	5.62	5.18	5.50	0.62
The GEK has increased the actor groups’ sense of belonging together	4.92	5.10	5.15	0.13
You feel as a part of the GEK	5.08	4.64	5.25	0.55
The GEK has increased the unified regional attitude towards the Canton or the Federal State	5.0	5.27	5.10	0.13
*Procedural criteria*				
The broad involvement of actor groups has greatly advanced the shared solution of the problem	5.57	4.64	5.45	2.11
All relevant topics have been discussed	5.15	5.36	5.15	0.10
So far neglected conflicts have been addressed	4.69	5.0	4.85	0.14
Actors attitudes and interests were respectfully considered	5.54	4.73	5.5	1.45
The communication was open and transparent	5.85	5.45	5.8	0.46
All participants have had the chance to contribute to the results	5.62	5.0	5.0	1.29
Important is to advance the interests of the actor group	5.07	5.00	5.35	0.25
Important is to advance the personal interests	3.71	4.09	4.50	0.39
Important is the consensus among the participants	5.07	5.82	5.60	0.36
Important are single compromises	4.86	5.36	5.55	0.42

Scale: 1 = Does not apply at all, 4 = neutral, 7 = Fully applies

*p < 0.05

With regard to the procedural criteria, the actors assessed the process as reasonably comprehensive, interactive, and fair. The slightly lower values related to the conflicts that were addressed indicate a slight criticism that controversial discussions had been avoided. The slightly higher significance of reaching consensus as compared to reaching compromises, however, suggests that actors endeavored to understand others’ points of views and to find shared solutions.

### Further outcomes of the GEK Hasli

The GEK provided three tangible outcomes:

- The technical concept on the future management of the regional rivers that was based on actors’ discussions and was finally approved by them was used as a basis for the strategic river plan of the Hasli region.
- At the end of the participatory process, actors agreed, against prevalent original skepticism against river restorations, on several projects of integrated river management, including flood protection, sediment management, and river restoration. The local river management corporations were mandated to immediately start with the detailed planning of these projects.
- Local river management cooperatives initiated regular meetings to increase regional collaboration.

### Diffusion of actors’ social learning to the wider regional public

The GEK Hasli and its activities were presented in two short articles in a regional newspaper (Berner Oberländer), and naturally, the public was also informed about this procedure via informal communication. The regional newspaper (5.12/7) and informal communication (4.92/7) were found to be the clearly most relevant information channels for regional residents to receive information about regional decisions.

Although the respondents who returned the questionnaire probably belonged to the best informed part of the regional population, only one of six noticed what had come out of the GEK Hasli (Table 5, 6). The scale of information level was used to measure the diffusion of actors’ social learning to the regional public through information. However, as expected, residents’ information-level appeared to depend on their social integration in the region. In particular, residents’ personal network was found to be strongly associated with their information level on the GEK Hasli. Therefore, these parameters had to be controlled when testing the diffusion of social learning.

**Table 5:** Frequencies of information-levels on the GEK Hasli as indicated by the respondents

What have you heard about the GEK Hasli?	Percentage
1: Nothing	28.2%
2: That it took place	21.0%
3: Who participated or what was it about	34.2%
4: What came out of it	16.5%

**Table 6:** Differences of social integration characteristics between the information-level categories based on ANOVA

Information level	1	2	3	4	F-value
I feel attached to the region	6.13	6.3	6.56	6.6	4.76**
Societies and other local institutions are important to me	5.21	5.67	5.74	6.07	5.51**
I have a good personal network here	4.65	5.37	5.35	5.85	10.58***
There are interest groups in the region that represent my concerns in public	4.43	4.61	5.0	5.15	4.34**

*p < 0.05; **p < 0.005; ***p < 0.001

Hierarchical regression analyses were conducted, with variables on effects of social learning included as (single) dependent variables, information-level as an independent variable, and 12 items measuring respondents social integration in the region as control variables. The results revealed three key insights. (1) Respondents’ information-level on the GEK Hasli only had a significant effect on very generalized social learning effects (e.g. that the GEK Hasli was an asset for the region). (2) Their information level had no significant effect on variables measuring any of the three elements of actors’ problem perspectives. (3) Strong perceived agreements with the three actor types’ positions, in particular nature protection types, had the strongest influence on social learning effects (Table 7).

**Table 7:** The influence of respondents’ reported information-level and further control variables on three effects of social learning based on hierarchical regression analysis

	Flood protection should always be combined with river restoration	The GEK Hasli is for the region an asset	Through the discussions on water management I have improved my problem understanding
Information-level	ns	0.18*	0.205*
Newspaper important for information on regional decisions	0.144*	ns	ns
Public information events important for information on regional decisions	ns	ns	0.165*
Agreement with hydropower actors	ns	0.206**	ns
Agreement with agriculture actors	− 0.162*	− 0.124*	ns
Agreement with nature protection actors	0.387***	0.361***	0.264***
The regional population has been involved in regional planning processes	ns	0.211*	ns
I have a good network in the region	0.09*	− 0.122	ns
I have grown up in the region	− 0.123*	ns	ns
*R2corr*	*0.127*	*0.356*	*0.233*

p < 0.005; ***p < 0.001

## Discussion

Recent literature suggests that social learning through deliberative processes provides a promising approach to enable the implementation of societal decisions and sustainable transition (Pahl-Wostl et al. 2007; Eriksson et al. 2019). Furthermore, social learning effects are in particular expected to evolve from reframing of conflicting actors’ problem perspectives that allow for finding shared solutions (Asah et al. 2012; Cundill 2012). There is, however, only very scarce empirical evidence that supports these conceptual assumptions. This study examined these assumptions by systematically evaluating a participatory process to elaborate an integrated regional water resource management concept (GEK Hasli) in the Swiss Alpine river catchment of the Hasli Aare using a quasi-experimental intervention research approach. The analysis of this evaluation provided robust empirical evidence on the social learning process through deliberative interactions of regional actors regarding baseline conditions, direct effects and diffusion or spillover effects.

### Pre-conditions for social learning

The findings demonstrated that the regional actors, and in particular the identified three actors types, held strongly diverging problem perspectives on the issue, which complicates the process of finding shared solutions. At the same time, however, regional actors appeared to have common interests such as the protection from natural hazards or the conservation of the landscape, which makes them aware of their interdependence and motivates them to search for shared solutions: thereby forming the basic condition for social learning (Ostrom et al. 1999; Pahl-Wostl et al. 2008). The ex-post survey furthermore confirmed that the involved actors managed to build up a group identity during the participatory process, which is expected to play a crucial catalytic role for actors to reframe their problem perspectives (Schusler 2003; Cundill 2012). Recent studies confirm that, due to these reasons, the regional level, rather than the local or the provincial level, is appropriate to approach natural resource management issues (Gailing and Rohring 2016; Müller et al. 2020).

### Effects of social learning

The comparison of the pre- and post-measurement provides robust evidence that actors substantially changed and reframed their problem perspectives during the participatory process, at least with regard to their self-reported appraisal. A general pro-environmental shift, and in particular a systematic convergence, could be determined in all elements of actor types’ problem perspectives. Among the three elements of actors’ problem perspectives, the effects of the social learning process, however, appeared to evolve differently. Only minor changes could be observed for most items that were used to evaluate the meanings actor types associated with the regional river environments. These meanings seem to belong to the aspects of actors’ problem perspectives that form their basic environmental knowledge. Therefore they are not, or only marginally, affected by framing processes due to regional conflicts, so that the participatory process could only produce minor changes in the sense of reframing. However, regarding the objectives, and in particular regarding the beliefs related to the water resource management, actor types’ problem perspectives considerably changed during the process. The strong, mainly converging changes in actor types’ assessments of water resource management related objectives indicate that they had revised, at least for some issues, their problem perspectives and thus achieved a double-loop learning level (Biggs 2011). The finding that the actors agreed on shared solutions at the end of the participatory process, and launched several projects of integrated river management, supports this insight. The even more substantial changes in actor types’ water resource management related beliefs that form part of actors’ value systems suggest that a triple-loop learning has also taken place. This is again supported by a concrete outcome of the participatory process: that local water management cooperatives decided to form a new common institution. However, the finding that the beliefs converged or polarized depending on the conflict field shows that this learning-level was only partly achieved.

Actors’ ex-post subjective assessment of the success of the GEK Hasli process substantiates the findings on actors’ reframing of their problem perspectives: in particular their positive assessment of the convergence of actors’ attitudes and their increased shared understanding of the problem. These assessments appeared to be slightly more positive than the ex-post assessments in the studies by Garmendia and Stagl (2010) and Ernst (2018). Interestingly, actors’ ex-post assessments of their personal changes of perspectives (only 20% positive) are much less positive than their general assessment, and also less positive than the assessment of the personal changes in Ernst’s study (2018: 43% positive). This difference can be explained by the different target groups, which in Erst’s study was the general population, and by the social desirability among actors to appear consistent (Buchecker et al. 2013).

### Diffusion of social learning effects

To our knowledge, this study is the first to measured the diffusion of social learning effects to the regional population through communicative processes. No evidence was found that actors’ reframing of their problem perspectives spilled over to the regional population who were not directly involved. The results of the regression analysis show that better informed respondents were more positive about the value added of the GEK Hasli and also assessed their problem understanding as more positive, which suggests that some generalized content of social learning has spilled over to the wider public. As the survey also revealed a rather limited information-level among the regional residents on the GEK Hasli, this finding is somewhat promising. It suggests that more intensive communication might enable a more substantial diffusion of social learning, which is needed if social learning processes are to promote sustainable transition.

### The dynamics of social learning

Overall, the evaluation of a participatory integrated regional water resource management process shows that involving relevant actors in deliberative discussion to negotiate the future development of a regional resource using an integrated, cross-sectional approach enables shared solutions to be found that would not materialize with a sectional approach, such as regional actors’ agreement to realize integrated river projects including originally unpopular river restorations. Opening the thematic boundaries enables the catalytic process of involved actors building a group identity based on shared interests. This focus on shared interests makes it easier for the actors to open their problem perspectives and the space for shared solutions thereby increases, which is for example expressed by actor groups’ stronger consensus about ensuring residual flow. The focus on problem perspectives in this study highlights, as suggested by Ostrom (1999), the key role of the shared interests, which normally exist in a regional context, in successful social learning processes as a basis for sustainable landscape and natural resource management.

### Limitations

In spite of the systematic research design and the robust evidence found, this study is not without limitations, which need to be considered. As in most evaluations of participatory processes, the sample size is certainly a critical point: even though it was higher in this study than in earlier studies. The comparison of not identical samples in the repeated measurement is more critical and some uncertainties about the comparability of the samples remain: even though the pseudo panel approach (Bernard et al. 2011) helped to cope with this issue and allowed for a better illustration of the learning process. Consistency with the subjective ex-post assessments of the process, as well as with pairwise comparisons of individual assessments before and after the process that could not presented in this study, however, support the validity of the findings. Regarding the diffusion of social learning, the findings based on a cross-sectional standardized survey only provide correlations and cannot substantiate causal relationships: that the information level really increased respondents’ problem understanding. Future research should evaluate participatory processes, in which the regional population is directly involved, and measure the problem perspectives of random samples of the regional population using a longitudinal research design. Directly involving the regional population might also contribute to mitigating a further issue of the study: the rather low response rate, which indicates a considerable degree of self-selection and an over-representation of better-informed residents. The poor public communication about the GEK Hasli was certainly a relevant justification for many residents not to participate in the survey. A final limitation refers to the case study design. Our findings are based on the evaluation of a single pilot project in a Swiss context, in which deliberative forms of democracy might be more familiar than in other countries. To extend their generalizability, international studies would be needed that compare social learning processes in diverse institutional contexts.

## Conclusions

Environmental policies, including those seeking to implement sustainable energy transitions, struggle to implement their goals due to conflicting interests. Social learning, through deliberative involvement of relevant actor groups to agree on a shared problem understanding and shared solutions, is considered a promising approach to overcoming these blocking conflicts. A number of studies, which have mainly used ex-post measurements have provided a tentative confirmation of the conceptual expectations. This study evaluated a real participatory decision process, with a systematic quasi-experimental intervention research approach that focussed on the change of actors’ problem perspectives, so could provide sound empirical evidence of the assumed mechanism of social learning in the context of regional natural resource management in Switzerland. The findings confirm Ostrom’s (1999) assumption that a diversity of perspectives on natural resource use exist in modern, functionally networked regions, but that shared interests provide a potential basis for collaboration. These results substantiate the proposition that deliberative negotiations among actors on the future use of regional water resources contribute to the convergence of actors’ perspectives on the issue as well as to tangible outcomes: in this case an agreement on integrated regional projects and a new institution for regional collaboration. Furthermore, the study provides first tentative evidence that some generalized aspects of actors’ social learning, in particular the perceived added value of the GEK Hasli for regional collaboration, have also diffused to regional residents who have only been involved in the process through indirect communication.

Furthermore, the findings highlight, more distinctly than suggested in the existing theoretical literature on social learning, the key role of shared interests in catalyzing the learning process by providing the basis for building a shared identity among involved actors. This key role of shared interests as a pre-condition for social learning has two basic implications: social learning requires actors’ awareness of their mutual interdependence, and it can only materialize if broader problems, rather than single conflicts, are negotiated. This means that social learning through deliberations can primarily unfold its strength in integrated regional strategic planning. However, it also emphasizes the challenge of overcoming the discrepancy between the poor institutional infrastructure of regions and their key role for sustainable transition. The approach of social learning therefore calls for the installation of new forms of regional strategic planning, such as with regional actor platforms, but also, as the example of the watershed management organizations in Canada (Medema et al. 2015) has shown, a fundamental change in environmental governance in terms of a balanced allocation of power and resources.

## Appendix

See Table 8

**Table 8:** Principal component analysis of respondents’ reported agreement with the attitudes and interests of involved actor groups, Eigenvalue > 1, explained variance = 79.4%, Rotation method: Varimax with Kaiser normalization, loadings > 0.5 in bold

	Components		
	Ecology	Agriculture	Hydropower
Actor group	Loading		
Regional government agencies	**0.797**	− 0.367	− 0.033
Hydropower companies	− 0.172	0.094	**0.806**
Farming corporations	− 0.260	**0.870**	0.228
Forestry corporations	0.045	**0.934**	0.121
Conservation associations	**0.629**	− 0.154	− 0.628
Tourism associations	**0.762**	0.358	0.361
Local municipalities authorities	0.084	0.319	**0.780**
Water management corporations	0.090	**0.640**	**0.539**
